# Impact of litter size on the hematological and iron status of gilts, sows and newborn piglets: a comparative study of domestic pigs and wild boars

**DOI:** 10.1186/s12917-024-03905-3

**Published:** 2024-02-23

**Authors:** Zuzanna Kopeć, Rafał Mazgaj, Rafał Radosław Starzyński, Xiuying Wang, Jolanta Opiela, Zdzisław Smorąg, Barbara Gajda, Jakub Nicpoń, Małgorzata Lenartowicz, Magdalena Ogłuszka, Mikołaj Antoni Gralak, Paweł Lipiński

**Affiliations:** 1 Department of Molecular Biology, Institute of Genetics and Animal Biotechnology, Polish Academy of Sciences, Jastrzębiec, Poland; 2 National Research Institute of Animal Production, Balice, Poland; 3 Department of Surgery, Faculty of Veterinary Sciences, Wrocław University of Environmental and Life Sciences, Wrocław, Poland; 4 Laboratory of Genetics and Evolution, Institute of Zoology and Biomedical Research, Jagiellonian University, Kraków, Poland; 5 Department of Physiological Sciences, Warsaw University of Life Sciences-SGGW, Warszawa, Poland

**Keywords:** Domestic pig, Iron deficiency anemia, Litter size, Pregnant sows, Wild boar (*Sus scrofa* L.)

## Abstract

**Background:**

The critically low hepatic iron stores of newborn piglets are considered to be a major cause of neonatal iron deficiency in modern breeds of domestic pig (*Sus domestica*). The main factor believed to contribute to this phenomenon is large litter size, which has been an objective of selective breeding of pigs for decades. As consequence, iron transferred from the pregnant sow has to be distributed among a greater number of fetuses.

**Results:**

Here, we investigated whether litter size influences red blood cell (RBC) indices and iron parameters in Polish Large White (PLW) piglets and gilts. Small and large litters were produced by the transfer of different numbers of embryos, derived from the same superovulated donor females, to recipient gilts. Piglets from large litters obtained following routine artificial insemination were also examined. Our results clearly demonstrated that varying the number of piglets in a litter did not affect the RBC and iron status of 1-day-old piglets, with all showing iron deficiency anemia. In contrast, gilts with small litters displayed higher RBC and iron parameters compared to mothers with large litters. A comparative analysis of the RBC status of wild boars (having less than half as many piglets per litter as domestic pigs) and PLW pigs, demonstrated higher RBC count, hemoglobin level and hematocrit value of both wild boar sows and piglets, even compared to small-litter PLW animals.

**Conclusions:**

These findings provide evidence that RBC and iron status in newborn PLW piglets are not primarily determined by litter size, and indicate the need to study the efficiency of iron transport across the placenta in domestic pig and wild boar females.

## Background

Early postnatal iron deficiency anemia (IDA) is a frequent disorder in mammals, but it only occurs regularly in domestic pigs [1, 2]. Hepatic iron reserves are the main source of this microlement to meet erythropoietic needs during early development [3]. It is not surprising therefore that critically low fetal/newborn hepatic iron stores, ranging between 50 and 70 mg iron [4], are considered a primary cause of IDA in suckling piglets. Moreover, considering the low iron concentration in the colostrum and milk of sows [4], the natural iron supply is clearly inadequate to satisfy iron requirements in rapidly growing piglets. For many decades, pigs have been bred for growth rate, and this has greatly increased their iron demand [1, 2, 5]. Large litter size and high birth weight of piglets are two further objectives of selective pig breeding that have very likely contributed to the paucity of iron stores [6]. In modern pig breeds, multiple gestations of more than 10 piglets are thought to exceed the physiological potential of sows to provide sufficient iron to their fetuses.

The aim of this study was to check whether the size of litters of Polish Large White (PLW) gilts influences red blood cell (RBC) indices and blood plasma iron parameters in both the gilts after farrowing and their 1-day-old newborn piglets. For this purpose we examined animals from small and large litters obtained by transfer of different number of embryos to recipient gilts as well as animals from large litters following artificial insemination. Since iron deficiency has not been reported in the offspring of wild boar, the ancestor of domesticated pigs, which typically have less than half as many piglets per litter compared to domestic pigs [7], another goal of this study was to compare the RBC and iron status of wild boars and PLW pigs from different sized litters. Our results challenge the well-established view that litter size in the domesticated pig determines RBC and iron status in newborn piglets. We also present the first experimental evidence that RBC status in wild boar sows and piglets is higher than that of domestic pigs, regardless of litter size.

## Methods

### Animal experimentation

#### Polish Large White pig

The experiment with PLW pigs was conducted at the Pig Experimental Station in Żerniki Wielkie, belonging to the National Research Institute of Animal Production (Balice, Poland). The sows and gilts were housed under standard conditions (70% humidity, temperature 22 °C) in gestation cages (2.2 × 0.65 × 1.8 m) with straw bedding. Females were placed in farrowing cages (2.4 × 3.4 m) on day 110 of gestation. Until parturition, females were offered the standard fodder for pregnant sows containing 120 mg Fe/kg, as estimated by flame spectrometry.

### Obtaining small and large pig litters through regulated embryo transfer (ET)

This procedure was performed as previously described [8]. Importantly, in this study, the term ‘large litter’ was used solely for the purpose of contrasting with small litters. Specifically, litters of 14 ± 2 and 12 ± 1 piglets, obtained by artificial insemination and embryo transfer, respectively, represent the average litter size in the PLW breed [9]. In contemporary pig production, litters with 18–20 piglets do occur, objectively qualifying as large litters [10–13]. However, the frequency of such large litters in PLW pigs is relatively low [9]. Moreover, achieving litters of 18–20 piglets through embryo transfer technology is challenging.

#### Preparation of embryo donors

To obtain small and large litters, embryos were collected from PLW females for transfer to recipient animals. The embryo donors were fifteen 6-month-old gilts weighing 90–110 kg that were superovulated by intramuscular injection of 1500 I.U. PMSG (Pregnant Mare Serum Gonadotropin; Folligon, Intervet, Boxmeer, The Netherlands). After 72 h, the animals were injected with 1000 I.U. hCG (Human Chorionic Gonadotropin; Chorulon, Intervet, Boxmeer, The Netherlands). On the oestrus day, these females were inseminated twice at 12-hour intervals with a standard dose of semen.

#### Obtaining embryos at the 2–4 cell stage

Embryos were obtained 72–74 h after hCG injection by flushing each oviduct with 20 ml of PBS supplemented with BSA at 38 °C. The collected fluid was screened for embryos using a stereoscopic microscope. The embryos were then transferred to PBS supplemented with 20% FCS and morphological evaluation was carried out.

#### Preparation of embryo recipients

The embryo recipients were 6-month-old PLW gilts weighing approximately 90 kg. These females were synchronized by intramuscular injection of 750 I.U. PMSG. After 72 h, they were injected with 500 I.U. hCG. Symptoms of heat were observed after a further 24 h. ET was performed surgically on the 2nd day after the synchronized heat. Under full anesthesia, embryos were introduced into the fallopian tubes. The number of embryos per recipient animal depended on the experimental group: group I – 6–8 embryos each; group II – 14–16 embryos each. The effectiveness of the transfer was assessed by ultrasound scans of the recipients on the 30th and 45th day after ET, and based on the number of live piglets born. Using regulated ET we obtained large litters from five gilts and small litters from seven gilts of 10 ± 1 and 4 ± 2 piglets, respectively. Two piglets from each gilt were used, based on their body weight (litter average).

### Obtaining large pig litters through artificial insemination (AI)

PLW piglets from large litters (14 ± 2 piglets) obtained from six 1-year-old sows by routine artificial insemination were also used. These piglets have been included in the study as control animals to rule out the potential effect of embryo transfer procedure on changes in RBC indices and iron parameters in newborn piglets. Two piglets from each sow were used, based on their body weight (litter average).

### Wild boars

The experiment with wild boars was conducted at the Centre for Research into Forests and Game Breeding of Wrocław University of Environmental and Life Sciences (Złotówek, Poland). Clinically healthy wild boars females captured in the forest of Trzebnica and Oleśnica County, Lower Silesian Voivodeship, were kept in semi-wild conditions on a forest farm monitored by 12 cameras that were used to track their behavior, particularly rutting, mating and preparations for farrowing. The farm was divided into three enclosures of approximately 1300 m^2^ each. Two females were placed in each enclosure and a male boar was introduced for the mating period (November to January). The animals had *ad libitum* access to water and were fed maize grain, potatoes and fresh forage daily. The ground allowed for burrowing and other natural wild boar behaviors such as mud baths and scratching against tree trunks. For the experiment, six 1-2-year-old gilts and sows weighing 60–80 kg and their offspring (2 piglets from each gilt/sow) were used, based on their body weight (litter average).

### Collection of blood, colostrum, and tissue samples

Before blood sampling, the wild boar females were premedicated by intramuscular injection (using a Palmer’s weapon) of a mixture of methomidine (0.03 mg/kg b.w.), ketamine (9 mg/kg b.w.) and midanium (0.2 mg/kg b.w.). Blood from PLW and wild boar gilts/sows was drawn 24 h after farrowing by venipuncture of the jugular vein (*Vena jugularis externa*). The samples were centrifuged (1500×*g*, 10 min, 4 °C) to separate the plasma. Plasma samples were immediately aliquoted and stored at -80 °C.

One-day-old PLW and wild boar piglets were premedicated with 4 mg/kg azaperone (Stresnil, Elanco; 40 mg/mL) by intramuscular injection and blood was drawn by cardiac puncture. Following blood collection, the animals were immediately euthanized by intracardiac injection of 0.5 mL/kg b.w. of Morbital (133.3 mg/mL of sodium pentobarbital + 26.7 mg/mL of pentobarbital; Biowet, Puławy, Poland).

To obtain colostrum samples (2–3 ml), PLW and wild boar females were hand milked into plastic tubes after ensuring the udder was clean.

Placental samples were collected from the maternal-chorioallantoic interface at each horn, immediately after its expulsion, then washed with PBS. Livers were excised from piglets after laparotomy. All tissue samples were immediately frozen in liquid nitrogen and stored at -80° C prior to analysis.

### Measurement of red blood cell (RBC) indices and blood plasma iron parameters

RBC indices were determined using an IDEXX ProCyte Dx, an automated hematology analyzer (IDEXX Laboratories Inc., Westbrook, ME, USA). Plasma iron level and plasma ferritin concentration were evaluated with a COBAS INTEGRA 400 plus biochemical analyzer (Roche Diagnostics, CH-6343 Rotkreuz, Switzerland).

### Measurement of iron content in placenta, liver and colostrum samples

The non-heme hepatic and placental iron content was determined by acid digestion of the samples at 100 °C for 10 min, followed by colorimetric measurement of an iron-ferrozine complex (absorbance at 560 nm, Beckman DU-68) as described previously [14].

A 20 µL sample of colostrum was diluted in 2 ml of boiling Suprapur-grade nitric acid (Merck, Darmstadt, Germany). The total iron concentration was then measured using the graphite furnace atomic absorption spectrophotometry (AAS) technique (AAnalyst 800, Perkin-Elmer, Waltham, MA, USA). Three samples of a standard reference material (197.94 ± 0.65 Fe mg/kg), were analyzed for normalization of the obtained data.

### Measurement of Hepcidin-25 levels in blood plasma

A commercially available sandwich enzyme-linked immunosorbent assay [DRG Hepcidin 25 (bioactive) HS ELISA Kit; DRG Instruments GmbH, Germany] was used for quantification of the hepcidin level in blood plasma.

### Statistical analyses

The results are presented as means ± SD (standard deviation). Before comparision of means, Levene’s test was performed for the assessment of the equality of group variances. For all presented results it was negative. One-way analysis of variance (ANOVA) was used for statistical evaluation of data and the Scheffe test (*p* < 0.05) was applied post-hoc.

## Results and discussion

Improvement of reproductive traits, including litter size has been one of the main goals of pig breeders over the last century. However, increasing the number of piglets per litter has raised questions concerning the biological limits of pregnant sows to provide sufficient amounts of nutrients to their numerous offspring. Indeed, iron deficiency is the most common disorder in the neonatal period in domestic pigs, resulting in severe iron deficiency anemia [1, 2]. Interestingly, no cases of iron deficiency have been recorded in the offspring of wild boar (*Sus scrofa* L.), the ancestor of domesticated pigs, suggesting that iron metabolism is well balanced in these animals. A possible explanation for this discrepancy is that during pregnancy, iron transferred from the domestic pig mother has to be distributed among typically more than 10 fetuses, compared to 4–6 in a wild boar sow.

**Fig. 1:**
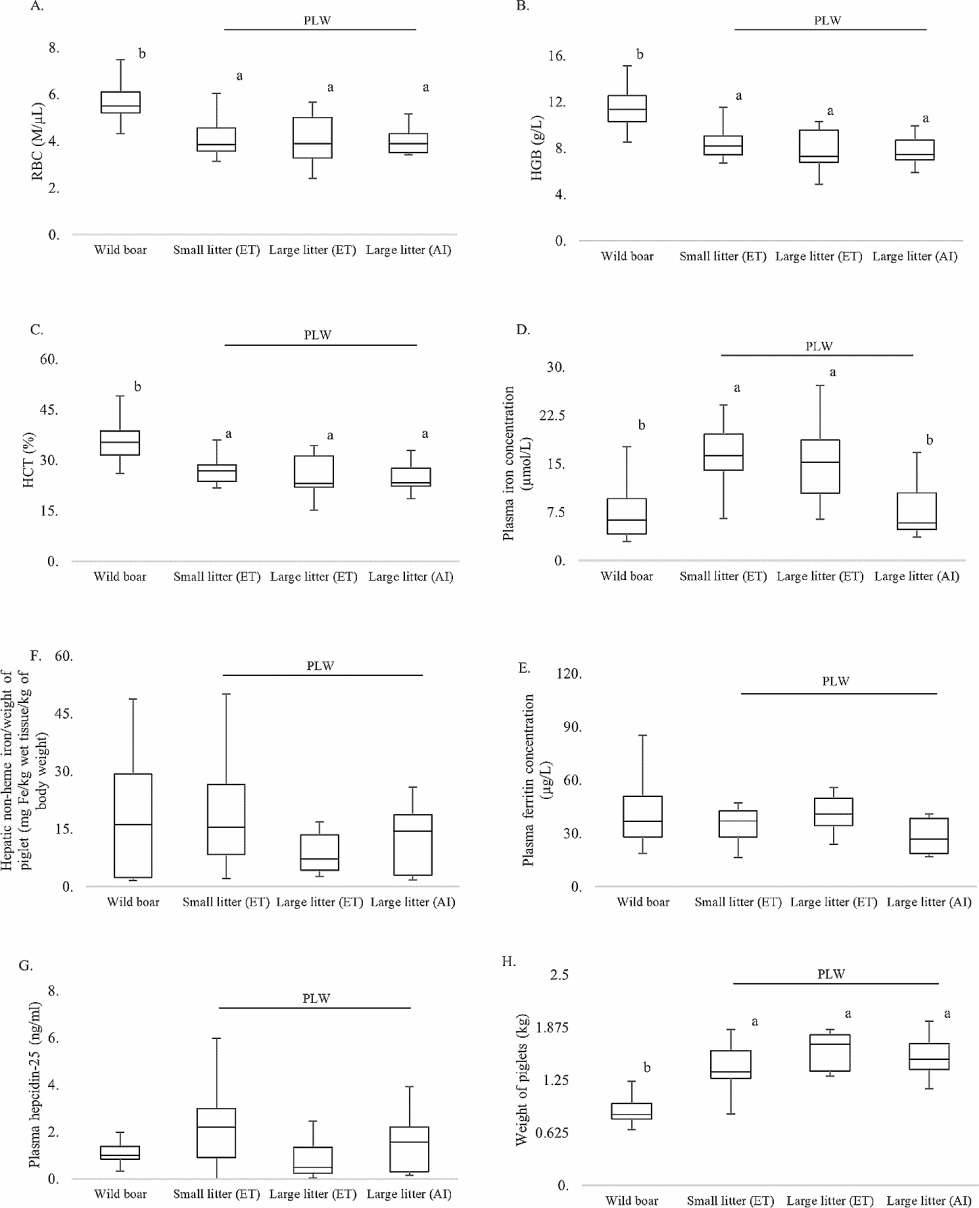
Red blood cell (RBC) and iron metabolism parameters in 1-day-old wild boar and PLW piglets from litters of different sizes. Piglets were allocated into four experimental groups: (i) Wild boar, *n* = 12; (ii) PLW small litter after embryo transfer (ET), *n* = 15; (iii) PLW large litter after ET, *n* = 10; (iv) PLW large litter after artificial insemination (AI), *n* = 12. (**A**) RBC count; (**B**) Hemoglobin (Hgb) concentration; (**C**) Hematocrit (Hct) value; (**D**) Blood plasma iron concentration; (**E**) Blood plasma ferritin concentration; (**F**) Relative hepatic non-heme iron content; (**G**) Blood plasma hepcidin-25 concentration; (**H**) Weight of piglet. Within each box, horizontal lines denote median values. Boxes extend from the 25th to the 75th percentile of each group’s distribution of values. Vertical extending lines denote adjacent values (i.e. the most extreme values within a 1.5 interquartile range of the 25th and 75th percentile for each group). Different letters indicate experimental groups between which statistically significant differences (*p* ≤ 0.05) were found

In this study, we attempted to clarify whether litter size is a factor affecting the RBC and iron status of 1-day-old Polish Large White (PLW) piglets and their mothers. Our results clearly demonstrated that PLW piglets from litters of various sizes showed similar (not statistically different) values of RBC indices that are indicative of IDA [15, 16], which contrasts with the higher, physiological RBC status of wild boar piglets (Fig. 1A–C). This suggests that pregnant domestic pig gilts, even those having a small number of piglets in the litter (4–6), are unable to provide across the placenta sufficient amounts of iron to meet the erythropoietic needs of their progeny. Surprisingly, the blood plasma iron levels detected in wild boar piglets were significantly lower than those of PLW piglets, irrespective of the litter size (Fig. 1D). This may be due to the greater dynamics of erythropoiesis in wild boar piglets, and thus the more vigorous uptake of transferrin-bound iron by erythroid progenitor cells in the bone marrow. Importantly, evaluation of hepatic iron reserves by both direct measurement of hepatic non-heme iron (Fig. 1F) and determination of blood plasma ferritin concentration (Fig. 1E) showed no statistically significant differences between wild boar and PLW piglets. Similarly, no differences were found in the plasma level of hepcidin (Fig. 1G), the liver-derived peptide that blocks iron efflux from iron-recycling macrophages in the liver, and from iron-storing hepatocytes [17]. Taken together, these results suggested that hepatic iron sources contribute to systemic iron availability to the same extent in both wild boar and PLW piglets.

**Fig. 2:**
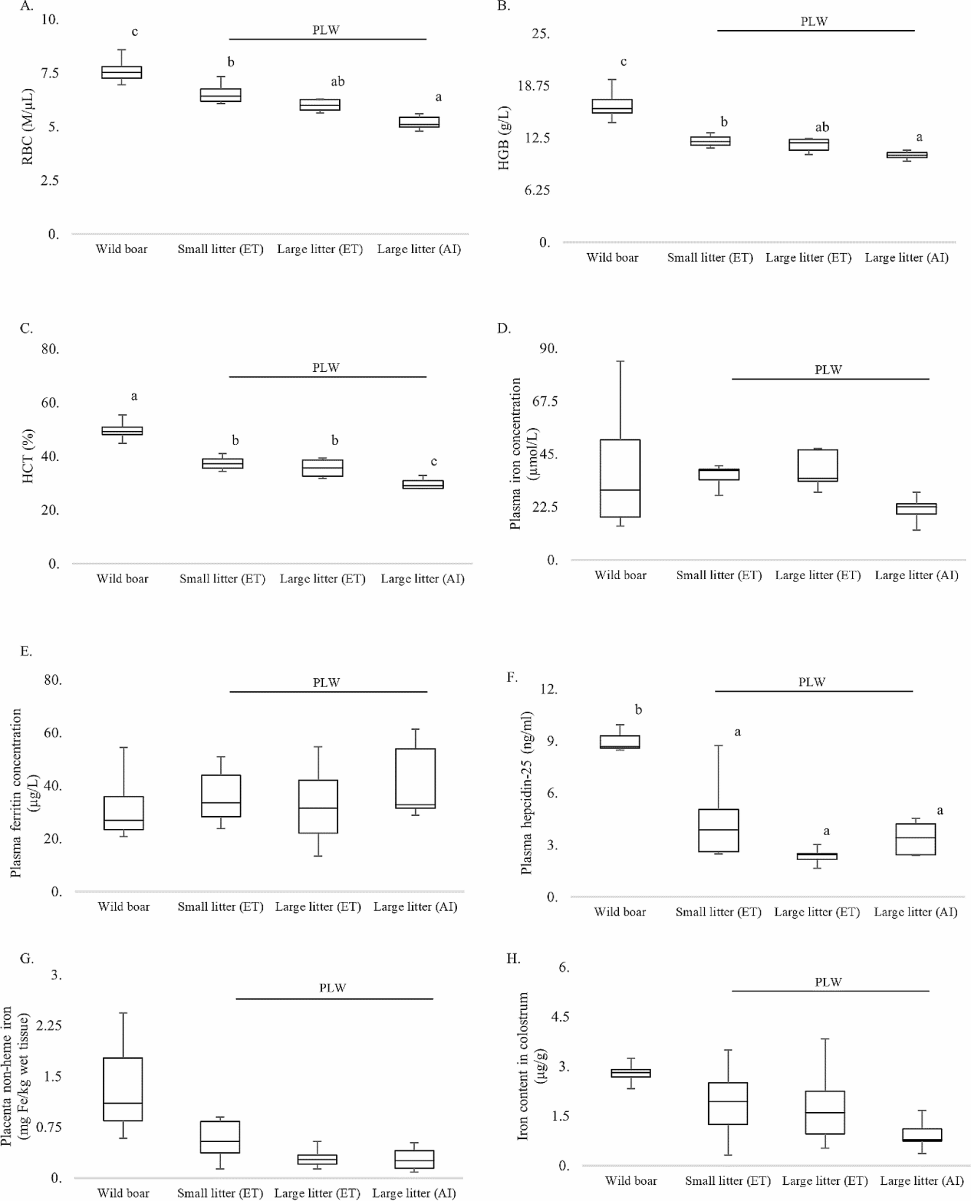
Red blood cell (RBC) and iron metabolism parameters in wild boar and PLW females with litters of different sizes. Females were allocated into four experimental groups: (i) Wild boar, *n* = 6; (ii) PLW small litter after embryo transfer (ET), *n* = 7; (iii) PLW large litter after ET, *n* = 5; (iv) PLW large litter after artificial insemination (AI), *n* = 6. RBC indices were determined for females from each group 24 h after farrowing: (A) Red blood cell (RBC) count; (**B**): Hemoglobin (Hgb) concentration; (**C**) Hematocrit (Hct) value; (**D**) Blood plasma iron concentration; (**E**) Blood plasma ferritin concentration; (**F**) Blood plasma hepcidin-25 concentration; (**G**) Placental non-heme iron content; (**H**) Total iron content in the colostrum. Within each box, horizontal lines denote median values. Boxes extend from the 25th to the 75th percentile of each group’s distribution of values. Vertical extending lines denote adjacent values (i.e. the most extreme values within a 1.5 interquartile range of the 25th and 75th percentile for each group). Different letters indicate experimental groups between which statistically significant differences (*p* ≤ 0.05) were found

As in piglets, the RBC status was also significantly increased in wild boar mothers compared to PLW gilts, including those having only 4–6 piglets per litter, i.e. equivalent to typical wild boar litters (Fig. 2A–C). It is unlikely that the higher RBC indices in wild boar sows evaluated ~ 24 h after farrowing are a consequence of greater nutritional iron availability during pregnancy, considering that these animals ingested iron mainly from the soil and corn grain, whereas PLW gilts received iron-enriched feed containing 120 mg Fe/kg in the form of FeSO_4_·H_2_O. It is, however, conceivable that the balanced RBC status of wild boar females (even when compared to PLW gilts with small litters) results from the smaller iron demand due to the much lower body weight of their newborn piglets (wild boar − 0.8 kg *versus* PLW − 1.4 kg, Fig. 1H) and the females themselves (60–70 kg *versus* 90–110 kg). It should be emphasized that although RBC status of PLW gilts from all experimental groups was decreased, it remained within the range of physiological values [18] and did not indicate IDA (Fig. 2A–C). Therefore, it is not surprising that the plasma iron parameters (iron and ferritin levels) were similar in both wild boar and PLW mothers (Fig. 2D–E). It is likely that the maintenance of this iron status in PLW gilts requires enhanced iron absorption from the diet as well as iron recycling by macrophages. It is tempting to propose that these two processes are facilitated by the significantly lower concentration of hepcidin in the plasma of PLW gilts compared to wild boar mothers (Fig. 2F). The equivalent iron status of wild boar and PWL females from all experimental groups is also reflected in the similar iron contents in the colostrum (Fig. 2H).

Intriguingly, we observed that, in contrast to PLW piglets, litter size significantly influenced basic RBC indices of PLW gilts. RBC count, Hgb concentration and Hct values were all significantly higher in gilts having a small number of piglets compared to those with large litters especially obtained following routine artificial insemination (Fig. 2A–C). In this context, it is worth noting that iron metabolism during pregnancy is adjusted to meet the essential iron requirements of the fetus at the expense of the mother [19–21]. This is why we hypothesize that the reduced RBC status of PLW gilts with large litters results from preferential iron transport across the placenta to satisfy the greater total iron requirements of numerous fetuses. It should be noted that we did not detrmine RBC and iron status of gilts before performing ET procedure. Potential differences between gilts having large and small litters could influence their parameters after farrowing. However, this seems unlikely considering that these recipient gilts were closely related, the same age, housed in the same conditions, and given the same diet. Anyway, the lack of these results could be a limitation of our study.

Our findings raise questions concerning the comparative efficiency of transplacental iron delivery in wild boar and domestic pig females. Considering that iron content in the placenta tends to be higher in wild boar compared to PLW females regardless of litter size (Fig. 2G), it is tempting to speculate that at least iron transport across the apical side of the placenta is more effective in the former animals. Experiments are in progress in our lab to evaluate the molecular potential of iron delivery across the placenta of females from all experimental groups used in this study.

## Conclusion

Large litter size, which has long been an objective of selective breeding of domestic pigs, was thought to limit the hepatic iron stores of newborn piglets, which contributes to iron deficiency anemia. We provide evidence that RBC and iron status in newborn PLW piglets are not determined by litter size. Furthermore, comparison of the RBC status of wild boar and PLW piglets, irrespective of litter size, shows the better performance of wild neonates. Our results demonstrate that litter size is not a critical factor responsible for the anemic state of newborn piglets, at least in PLW breed.

## Data Availability

All data analyzed during this study are included in this manuscript. The raw data are available from the corresponding author on reasonable request.

## Abbreviations

PLW: Polish Large White
PMSG: Pregnant mare serum gonadotropin
hCG: Human chorionic gonadotropin
PBS: Phosphate buffered saline
BSA: Bovine serum albumin
FCS: Fetal calf serum
ET: Embryo transfer
AI: Artificial insemination

## References

[1] Svoboda M, Drábek J (2005). Iron Deficiency in Suckling piglets: Ethiology, clinical aspects and diagnosis. Folia Vet.

[2] Szudzik M, Starzyński RR, Jończy A, Mazgaj R, Lenartowicz M, Lipiński P. Iron supplementation in suckling piglets: an ostensibly easy therapy of neonatal iron deficiency anemia. Pharmaceuticals. 2018; 11:128. 10.3390/ph11040128 PMC6315738 30467279

[3] Gambling L, Czopek A, Andersen HS, Holtrop G, Srai SKS, Krejpcio Z (2009). Fetal iron status regulates maternal iron metabolism during pregnancy in the rat. Am J Physiol - Regul Integr Comp Physiol.

[4] Venn JAJ, McCance RA, Widdowson EM (1947). Iron metabolism in piglet anaemia. J Comp Pathol Ther.

[5] Kim JC, Wilcock P, Bedford MR (2018). Iron status of piglets and impact of phytase superdosing on iron physiology: a review. Anim Feed Sci Technol.

[6] Mahan DC, Watts MR, St-Pierre N (2009). Macro- and micromineral composition of fetal pigs and their accretion rates during fetal development. J Anim Sci.

[7] Fernández-Llario P, Mateos-Quesada P (1998). Body size and reproductive parameters in the wild boar Sus scrofa. Acta Theriol.

[8] Poniedziałek-Kempny K, Gajda B, Rajska I, Gajda L, Smorąg Z (2020). Piglets produced by transfer of embryos obtained by in vitro fertilization of oocytes matured in vitro with thymosin: a case report. Medycyna Weterynaryjna.

[9] Nowak B, Mucha A, Moska M, Kruszyński W (2020). Reproduction indicators related to litter size and Reproduction cycle length among sows of breeds considered maternal and Paternal Components kept on medium-size farms. Animals.

[10] Björkman S, Oliviero C, Rajala-Schultz PJ, Soede NM, Peltoniemi OAT (2017). The effect of litter size, parity and farrowing duration on placenta expulsion and retention in sows. Theriogenology.

[11] Kemp B, Da Silva CLA, Soede NM (2018). Recent advances in pig reproduction: focus on impact of genetic selection for female fertility. Reprod Domest Anim.

[12] Thorsen CK, Schild S-LA, Rangstrup-Christensen L, Bilde T, Pedersen LJ (2017). The effect of farrowing duration on maternal behavior of hyperprolific sows in organic outdoor production. Livest Sci.

[13] Oliviero C (2023). Offspring of hyper prolific sows: immunity, birthweight, and heterogeneous litters. Mol Reprod Dev.

[14] Torrance J, Bothwell T (1980). Tissue iron stores. Iron (methods in Hematology).

[15] Egeli AK, Framstad T, Morberg H (1998). Clinical Biochemistry, Haematology and Body Weight in piglets. Acta Vet Scand.

[16] Kegley EB, Spears JW, Flowers WL (2002). Iron methionine as a source of iron for the neonatal pig. Nutr Res.

[17] Nemeth E, Ganz T. Hepcidin-ferroportin interaction controls systemic iron homeostasis. Int J Mol Sci. 2021;22. 10.3390/ijms22126493 PMC8235187 34204327

[18] Oven IG, Svete AN, Hajdinjak M, Plut J, Stukelj M. Haematological profiles of pigs on different farms. Reflect Their Health Status. 10.21203/rs.3.rs-644732/v1.

[19] Gambling L, Lang C, McArdle HJ (2011). Fetal regulation of iron transport during pregnancy. Am J Clin Nutr.

[20] Sangkhae V, Fisher AL, Wong S, Koenig MD, Tussing-Humphreys L, Chu A (2020). Effects of maternal iron status on placental and fetal iron homeostasis. J Clin Invest.

[21] Mazgaj R, Lipiński P, Edison ES, Bednarz A, Staroń R, Haberkiewicz O (2021). Marginally reduced maternal hepatic and splenic ferroportin under severe nutritional iron deficiency in pregnancy maintains systemic iron supply. Am J Hematol.

